# Governing the commons beyond harvesting: An empirical illustration from fishing

**DOI:** 10.1371/journal.pone.0231575

**Published:** 2020-04-23

**Authors:** Xavier Basurto, Abigail Bennett, Emilie Lindkvist, Maja Schlüter

**Affiliations:** 1 Duke University Marine Lab, Nicholas School of the Environment, Duke University, Beaufort, NC, United States of America; 2 Department of Fisheries and Wildlife, Michigan State University, East Lansing, MI, United States of America; 3 Stockholm Resilience Centre, Stockholm University, Stockholm, Sweden; Universitat Autonoma de Barcelona, SPAIN

## Abstract

Harvesting has received most theoretical, empirical, and policy attention towards understanding common-pool resource dilemmas. Yet, pre-harvesting and post-harvesting activities influence harvesting outcomes as well. Broadening the analytical focus beyond harvesting is needed to imagine new ways of theorizing and governing the commons. Fishing—which is synonymous with harvesting—is a case in point. We contribute to a beyond-harvesting research agenda by incorporating concepts from common-pool resources theory that have not received enough attention in the literature. We compare two ubiquitous self-organizing strategies (i.e., fishing cooperatives and patron-client relationships) fishers use to access means of production and analyze their effects on the distribution of benefits resulting from harvesting. We use rarely available longitudinal data of monetary loans to fishers in Mexican small-scale fisheries and find that cooperatives can deliver broader distribution of benefits than patron-client relationships. Our study highlights the importance of historically and contextually situating analyses linking the effects of pre-harvesting processes on harvesting outcomes, and the benefits of broadening the scope of inquiry beyond a narrow policy attention on harvesting to move towards a fuller understanding of commons dilemmas.

## Introduction

Understanding how to govern harvesting of common-pool resources has been a central concern for the development of common-pool resources theory [1]. In certain types of commons like fisheries, the label ‘fishing’ is synonymous with harvesting. The image of men on a boat on the water is immediately evoked when thinking about fishing. Yet fishing starts at home cooking the food needed for the outing, or in the negotiation for access with authorities, or with those in control of the fishing means of production and commercialization channels. Indeed, although harvesting is only one aspect informing how users structure and govern their interactions, regulations tend to focus on issues related solely to harvesting (i.e., regulating access to harvesting or harvesting methods). Is this policy-making focus on harvesting at the expense of pre- or post-harvesting well warranted? We hypothesize that it is not, and the goal of this paper is to offer avenues towards broadening the scope towards a 'beyond-harvesting' research agenda for common-pool resources and policy analysis.

The overarching attention to harvesting is evident in a recent inductive discourse analysis of the small-scale fisheries literature drawing from a database of 1,723 peer-reviewed papers [2]. As for governance attention between pre-harvesting and post-harvesting, post-harvesting seems to be receiving a bit more attention, particularly in relation to processing of catch, markets, and trade [3]. Yet questions regarding how fishers’ strategies to access fishing means of production affect harvesting outcomes remain of central theoretical and policy relevance but seem to have received less attention. For the above reasons, in this paper we focus our empirical analysis on addressing this question, specifically, linking how small-scale fishers’ strategies to access fishing means of production, property rights (e.g., fishing permits), and credit, which is often done through patron-client relationships or fishing cooperatives affects their distribution of economic benefits. Overall, we argue that taking a beyond-harvesting approach expands our ability to ask better questions about why common-pool resources appropriators in general, and fishers in particular, harvest or fish the way they do, which ultimately might provide policy-makers with additional regulatory levers to steer management and improve common-pool resources governance [4]. In line with much of the common-pool resources literature, the focus of this paper is on understanding the institutions that comprise governance. Institutions are the prescriptions that humans use to organize all forms of repetitive and structured interactions including those within families, neighborhoods, markets, firms, sports leagues, churches, private associations, and governments at all scale” [5]. As prescriptions, institutions may be written or unwritten and include not only enforceable rules, but also the norms and even shared strategies that structure behavior and decision making. In the context of this paper, therefore, we use the term governance to refer to the sets of institutions that structure fishers’ interactions [5]. Importantly, as Ostrom [6] among many others highlighted, governance can be carried out not only by government authorities, but by a multitude of actors, including resources users themselves, frequently operating in the absence of government authority, thus the term self-governance.

Theoretically, we call attention to the concepts of ‘constitutional choice’ [7] and 'chains of action situations' or 'networks of adjacent action situations' proposed in the past [8]. These concepts provide an analytical lens for analyzing connections of influence between pre-harvest and harvest activities and among different arenas of decision-making. In applying these concepts in the context of small-scale fisheries, we reveal informal dimensions of constitutional choice, which are rarely—if ever—examined. In the remainder of the paper we describe the theoretical background in more detail. Next, we ground our discussion in the Mexican commercial small-scale fisheries context, comparing two opposing self-governance strategies to gain access to fishing means of production: fishing cooperatives and patron-client relationships, to illustrate how pre-harvesting decisions influence governance of harvesting activities. Our empirical data shows that fishers’ choice of strategy for accessing fishing means of production strongly influences the distribution of harvesting benefits. We conclude by exploring the implications of our findings for future common-pool resources theorizations and governance of small-scale fisheries more broadly.

## Theoretical background

The iconic work of Garret Hardin [9] brought attention to the regulatory importance of harvesting in common-pool resources settings and has had tremendous policy impact around the world [1]. Equally influential was the theoretical and policy-relevant work on fisheries economics by Gordon [10] and Scott [11], who called attention towards harvesting dynamics and the tragedy of the commons that would ensue without attention to harvesting controls. Dominant conceptualizations of policy tools for fishing, generally fall into three broad categories: output or catch controls; input or effort controls; and technical measures [12]. Yet, a careful review of the common-pool resources literature would show the field has encouraged broad attention to the institutional, cultural, and biophysical elements that interrelate to create patterns of production, not only appropriation or harvesting. For instance, Elinor Ostrom’s well-known design principles for long-enduring common-pool resources [6] encompass this broader focus. Some of the design principles refer to processes that take place pre-harvesting, such as the need for clearly defined (social and biological) boundaries of the common-pool resource itself, or the need to have collective-choice arrangements in place where individuals affected by the operational rules can participate in modifying those rules. Other design principles refer to processes that take place post-harvesting such as having in place conflict-resolution mechanisms or graduated sanctioning procedures so that fines or penalties match the magnitude or frequency of the offense.

We argue that increased attention to the concepts of constitutional levels of analysis and linked action situations, two different conceptual devices anchored on institutional analysis and the Institutional and Analysis Development (IAD) Framework, would encourage analysis of common-pool resources governance beyond harvesting. Herein, following the IAD Framework the action situation constitutes the focal unit of analysis, and is defined as the situation where "two or more individuals are faced with a set of potential actions that jointly produce outcomes" [5].

We bring back attention to the ‘constitutional level’ of analysis, a component of Ostrom’s well-known levels of analysis scheme [5] that has not received sufficient attention in the common-pool resources literature, particularly in informal settings. The second conceptual device follows McGinnis [8] by identifying a core set of linked actions situations characteristic of commercial common-pool resources like small-scale fishing. Conceptualizing small-scale fisheries as composed of an array of linked action situations offers a straightforward way to move beyond powerful imaginaries of fishing as harvesting, which obscure considerations of activities and decisions that take place before and after harvesting, yet are inherently important in understanding how harvesting is governed [3].

### Constitutional choice in informal settings

**Fig 1:**
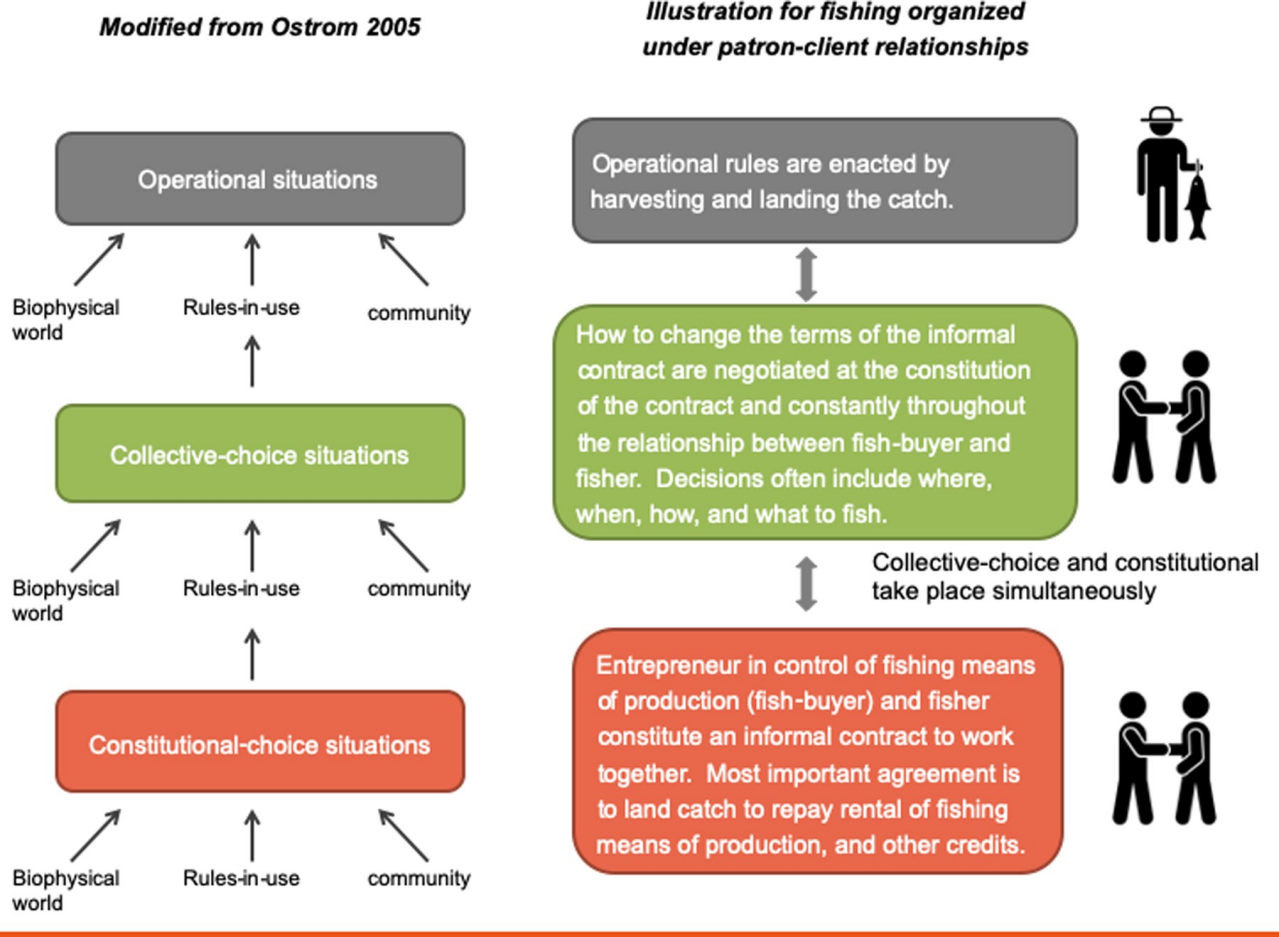
Levels of analysis adapted to the context of small-scale fishing using patron-client relationships as an example.

Back in the 1980s Vincent and Elinor Ostrom offered three distinct arenas levels as lenses under which collective action could be analyzed: the operational, collective-choice, and constitutional levels of analysis [7]. The operational level is the arena where daily decision-making processes that allow for harvesting activities take place. In the collective-choice arena decision-making processes determine operational rules; and in constitutional-level arenas decision-making for the design of collective-choice processes take place (Fig 1). Constitutional choice action situations are conceptualized as “the processes through which collective choice procedures are defined including legitimizing and constituting all relevant collective entities involved in collective or operational choice processes” [8].

The concept of an arena does not imply a formal setting [5]. While constitutional arenas are often exemplified through formalized bodies such as courts or legislatures this need not to be the case. Constitutional-choice arenas occur whenever individuals come together to constitute themselves as a group and in the process determine the fundamental rules about how the organization will be governed (however informally) and how future collective choice decisions will be made regarding who can participate, their basic rights, and responsibilities [7]. Furthermore, in many instances the same individual might be involved in constitutional, collective-choice, and operational situations [5]. This view of constitutional-choice arenas is most useful for the case of small-scale fishing where most interactions take place outside formal settings. Here, the constitutional level is often not far removed from the operational arena because it is the process itself of constituting, forming, or starting an interaction between fishers, or fishers and a fish buyer or patron—who will provide them with fishing means of production. The ‘constitutional’ layer provides information about the informal contractual arrangements, norms, and strategies underpinning group formation and thus influencing the structure of operational and collective choice rules and activities. As such, it also provides information about the motivations that individuals find to come together to transact with each other in the first place, as formal or informal as their association might be. In sum, we argue that by more explicitly incorporating constitutional-choice arenas and activities into notions of fishing, we effectively start moving away from the predominating notion of fishing as synonymous with harvesting.

### Self-governance arrangements in networks of action situations

The notion of linked action situations [8] is another theoretical device from common-pool resources theory useful to help us move away from centering the governance of common-pool resources solely on harvesting activities. Recall that an action situation is conceptualized as a unit of analysis in which at least two individuals (acting on their own or as agents of organizations) observe information and make decisions about which actions and interactions to engage with and their potential outcomes [5]. From Ostrom’s [5] perspective, all action situations are constituted by a common set of elements, such as (a) participants involved, and the (b) positions they adopt, the (c) actions they undertake, (d) information available about outcomes that the actions are likely to produce, the (e) control they have over actions and potential outcomes, and the (f) costs and benefits they can foresee of their perceived alternatives [5]. Under this perspective the great diversity of action situations we see in the world is a combination of the complex and almost infinite ways in which these elements manifest themselves and can be combined.

**Fig 2:**
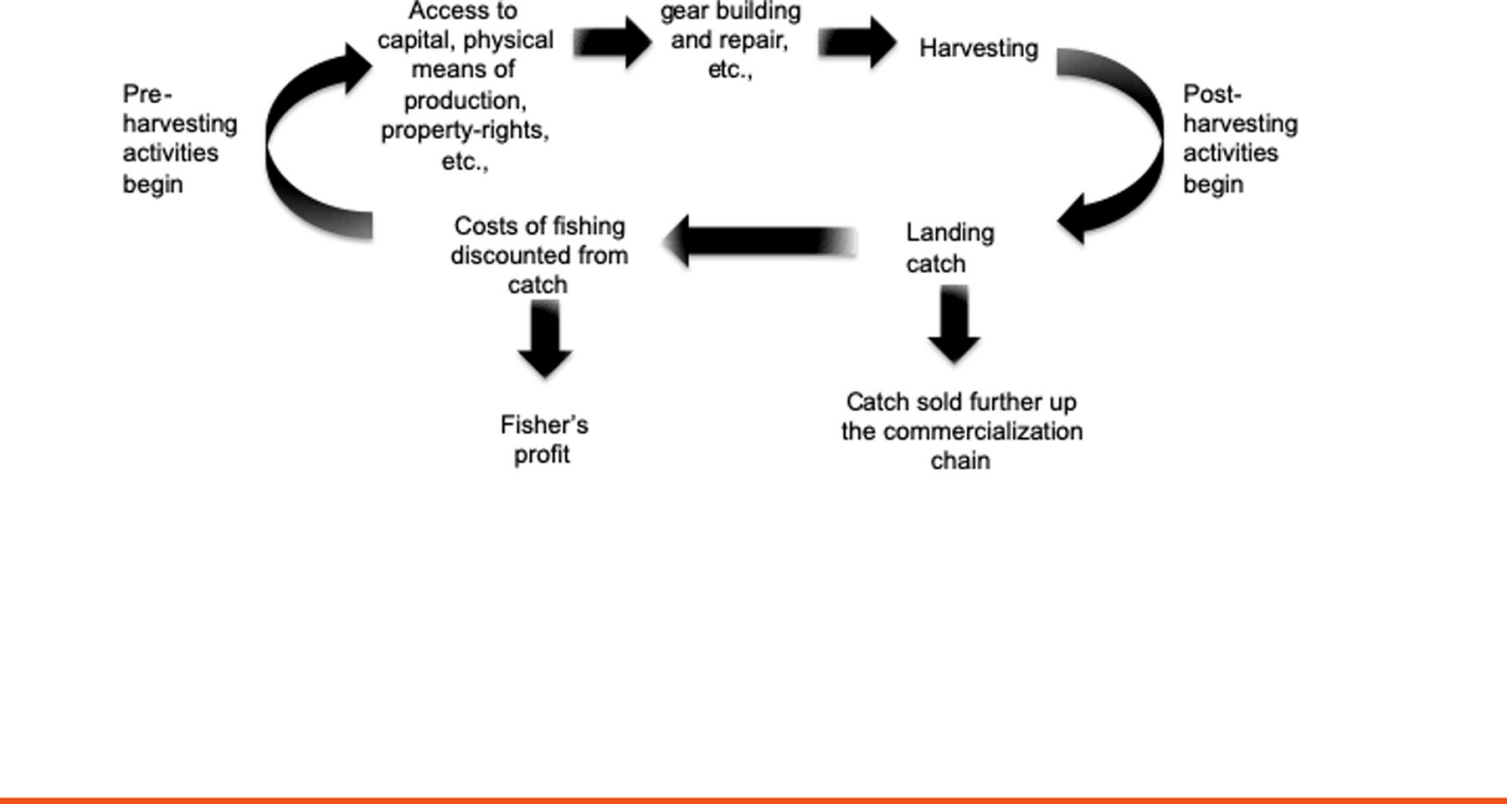
Governing the commons beyond harvesting. Governing the commons beyond harvesting requires incorporating pre-harvesting and/or post-harvesting into analyses of harvesting action-situations. The pre and post-harvesting action situations shown here are not exhaustive. This circular representation highlights the repetitive nature of a ‘typical’ fishing event with implications for the development of trust among fishers and fish buyers. Not all of these action arenas take place every day, nor are networks of action situations necessarily linked in a linear fashion as the diagram could be interpreted.

Using this conceptual lens one can view small-scale fisheries as composed of multiple linked action situations that can be categorized broadly as pre-harvesting, harvesting, and post-harvesting action situations. In Fig 2 we present a basic network of action situations common in commercial small-scale fishing that any observer of fishing activities in the field will be familiar with regardless of the particular way fishers associate with one-another, or the contextual setting in which they may be embedded. Fig 2 is presented as a circular representation to highlight the repetitive nature of fishing action situations, with implications for the development of trust-based relationships among fishers and fish buyers. Yet we also recognize fishers are confronted daily with networks of action situations linked to each other in complex and non-linear ways [13].

A complete analysis of all action situations in Fig 2 would require book-length treatment. Here we only discuss the action situation where actors negotiate access to capital, physical fishing means of production, and property-rights as a way to illustrate the crucial role pre-harvesting constitutional-choice arenas have in establishing relationships between fishers and fish buyers and the resulting harvesting behaviors we observe in operational arenas.

As in other common-pool resource settings there are a variety of strategies resource users can deploy to access capital, physical means of production, and fishing property rights (e.g. fishing permits). In the simplest possible configuration, a fisher owns his own boat and gear and harvests fish for household consumption or local sale. In contemporary small-scale fisheries it is less and less common for a fisher to be in control of all inputs needed for fishing. Increasingly fishers need to access these inputs from an entrepreneur, often the fish buyer to whom fishers sell their catch. In Mexico fishers can also access fishing means of production, property rights, and capital by gaining membership to a fishing cooperative, which will rent or loan these goods in exchange for catch. This is also the case if fishers are working on their own through verbal short-term contracts with a fish buyer (i.e., a patron-client relationship), and fishers often need to access capital to pay for short-term expenses (i.e., gas and food for the fishing trip) or pay rent for the use of the boat and fishing gear.

For the purposes of this paper cooperatives and patron-client arrangements are similar in that fishers receive capital, property rights, and inputs for fishing from a corporate agent (cooperative or patron), which binds them to land their catch with that same entity as a way to pay back for the received inputs. This is most common in contexts where the State under-provides legal, political, and economic resources necessary for small-scale fishers to participate in commercial harvesting activities.

We define a fishing cooperative as “an autonomous association of persons united voluntarily to meet their common economic, social, and cultural needs and aspirations through a jointly-owned and democratically-controlled enterprise” [14]. In contrast a patron-client arrangement is a relationship where capital, financial resources, property rights (when they exist) belong to a patron, fishers do not join in collective activities [15], and commercialization is often controlled by the patron [16]. Johnson [17] describes this form of self-governance as “common economic arrangements […] that link powerful individuals with numerous subordinates. In exchange [for] favors, including loans, protection, or intermediation, patrons receive labor, goods, political support or other benefits.”

These two forms of organizing fishing and accessing means of production, capital and property-rights are thought to be globally ubiquitous. In Mexico, officials estimate the existence of more than 3200 cooperatives [18]. In Turkey, one in every four fishers belongs to a cooperative [19] and more than 620 fisher’s syndicates are reported in Chile [20]. Patron-client informal arrangements can underpin the global seafood trade of certain species. For instance, the mahi mahi fishery of Peru and Ecuador, two of the most important producers in the world, is mostly small scale and based on informal, unwritten, trust-based contracts between fishers and fish buyers. With nearly 60% of their catch exported to the US, its estimated worth was 232 million US dollars in 2012 [21]. They are also prevalent in more localized fisheries and associated markets [22–23]. While patron-client relationships and cooperatives can be seen as two very opposing forms of organizing fishing, often gradients can be found in between these two [24–25]. Similarly, however, patron-client relationships and cooperatives are often the main pathways for low-income individuals in rural, developing country contexts to secure their livelihoods through gaining access to capital to afford the upfront costs of fishing trips (e.g. gas, bait, and food), fishing means of production (e.g., boat, motor, or fishing gear), property rights to the fishery (e.g., fishing permits), as well as to cash for personal loans (e.g., payment of corner-store bills, health emergencies, etc.) [4, 26]. It is well known that in rural developing country contexts, access to credit plays a key role in rural inhabitants’ ability to secure better livelihoods [27].

Differently from cooperative arrangements, patron-client relationships do not suffer the transaction costs of organizing and maintaining collective action. However, both are similar in that a corporate agent provides the fisher with the fishing inputs in return for the obligation to land catch and thus, assure the repayment of fishing inputs. Given that small-scale fishing mostly operates in a context of highly incomplete and difficult-to-enforce contracts they both suffer the problem of free riding and the likelihood of repayment is directly related to the quality of the relationship (e.g., trust) the agent has with the fishers.

### Historically situating our empirical analysis

How did cooperatives and patron-client relationships become dominant ways of organizing small-scale fishing in Mexico? While much of the day-to-day activities of small-scale fisheries take place largely outside of the reach of formal State authority, national, and international political economic processes have nonetheless shaped the contemporary context within which small-scale fishers navigate their livelihoods. Over time, States as well as international organizations have promoted different modes for distributing fishing property rights, capital, and commercialization with a variety of implications for how individual fishers engage in self-governance arrangements at localities.

In Mexico a long and continuing history of cooperatives and collectivism across economic sectors coupled with recent transitions in the 1990s away from State interventionism toward private sector investment has given rise to a context in which fishing cooperatives and patron-client relationships coexist and compete for labor, market power, and fisheries resources [28]. During the first half of the 20^th^ century, fisheries development in Mexico focused on the promotion of social interests and equitable distribution of resources [29–31]. In this regard, cooperatives were a key tool for organizing fishing labor, vying for partisan loyalty of rural populations, commercializing fish production, and distributing subsidies, infrastructure, and capital, as Mexico sought to ramp up fisheries production and modernize its fleet. During this period, cooperatives also enjoyed preferential access to valuable fisheries resources, codified in fisheries law [32].

Beginning in the late 1980s, State support for fishing cooperatives subsided, a symptom of a broader shift toward financial deregulation and decentralization driven in part by the debt crisis and foreign debt servicing obligations. This period saw reduced subsidies to fishing cooperatives and the dissolution of the parastatal firm marketing fisheries products and the State fisheries development bank [32]. The formal legal framework shifted concurrently. Amendments in 1992 to Mexico’s constitution made inshore resources available for private sector fishing permits through competitive bidding processes [28] and the 1994 fisheries law eliminated cooperatives’ exclusive access to some valuable species [32]. These changes opened opportunities for individuals and private firms to play a more prominent role in the control of capital and property rights in fisheries as well as the commercialization and marketing of fisheries products.

The broader legal and political context does not provide a full picture either, particularly when trying to understand how each of these arrangements performs in relation to key issues such as access to capital, fishing means of production and property-rights. Given that both institutional arrangements are subject to free riding from their members (i.e., not fulfilling informal contracts), understanding how loyalty/trust/reliability emerge and are maintained are central to understand their performance. We argue that bringing such understandings to common-pool resources theory is central to broadening conceptualizations of fishing governance beyond harvesting, and to do this it is necessary to incorporate notions of constitutional choice.

### Using the concept of constitutional choice to understand group formation

Applying this concept in the context of small-scale fisheries reveals informal dimensions of constitutional choice that have not received much attention in the common-pool resources literature. In this context, fishers and fish buyers enter into a constitutional choice situation when they first decide to associate with each other to conduct the business of fishing. This can take place in the context of a fishing organization e.g., a fishing cooperative, or a patron-client relationship. This is a constitutional choice situation because the rules and norms structing how they will establish the terms of access to capital, fishing means of production, price of catch, etc., between the fisher and fish buyer are determined at this time, as well as how these terms can be modified (Fig 1).

In return for providing fishing inputs to the fisher, the cooperative or fish buyer expects the landing of catch at the agreed-upon price and the discount of the upfront loan to buy bait, fuel, food, from the fisher’s profit. Given that these are hard-to-enforce contracts (often verbal), there is always a risk fishers will fail to meet the obligation to land catch from which the loan is paid. This risk is particularly high in contexts of high local price competition and/or where a fisher can land his catch elsewhere in order to avoid repaying the loan or rent without facing (legal) consequences besides those resulting from direct cooperative or fish buyer action. In the long run, however, meeting obligations may be of interest to a fisher who wishes to continue to have access to personal loans, capital, gear, and fishing permits through subsequent transactions. In any case, because the possibility of such behavior is present, cooperatives and fish buyers face strong incentives to identify and associate with fishers they can trust in order to increase the likelihood to attain a steady supply of fish and return on investment.

It is well established that trust and relationship-building has a key influence on the persistence and success of buyer-seller relationships [33–35]. Particularly for the constitution of groups in constitutional-choice arenas where legal enforcement mechanisms are scarce or costly [36–37], such as in small-scale fishing contexts.

To illustrate the importance of incorporating constitutional choice action situations that take place before harvesting into governance analysis about harvesting, we take a look at these issues in the context of a fishing cooperative and patron-client relationship. This comparison illustrates similarities and differences between the two forms. First, we examine a patron-client structure where the patron has significantly more capital than fishers and is in control of fishing property rights, means of production, and access to the market. Next, we examine a cooperative, which in our case is a well-functioning one in the sense that it is able to keep basic record-keeping about catches and lending to its members, among other measures of successful collective action.

## Methods

To highlight the importance of the role of constitutional choice in the formation of seller-buyer groups such as cooperatives and patron-client relationships in the informal context of small-scale fishing, we took two complementary approaches: First we investigated how fish buyers selected the fishers with whom to transact with by conducting in-depth interviews with fish buyers (this issue has been much more explored in the formal contexts of cooperatives and so it is not included here). Second, we assembled two longitudinal databases related to the provision of loans. One comprises data from patron-client interactions and the reciprocating behavior of fishers and the other from a fishing cooperative and the reciprocating behavior of fishers.

For the involvement of human subjects in this research we obtained Institutional Review Board (IRB) approval at Duke University (#A0694) and the need for written consent from interviewees was waved. Oral consent was obtained from participants at the beginning of the interviews. During this time participants were briefed the data would be used for research purposes and about the voluntary nature of their participation. Interviewees were encouraged to express any questions and concerns at any moment during the interview and knew they could withdraw at any time. Interviews took place one-on-one in a setting of the participant’s choice.

All personal information contained in the longitudinal databases was anonymized. Names were replaced with a code that cannot be traced to the original name. Original data is securely stored and personnel conducting analysis have no access to the original data. Our research did not need to be reviewed and approved by a Research Ethics Committee in Mexico.

### Who to work with?

To understand how fish buyers and fishers entered into constitutional choice arrangements with one another, we needed to know what criteria fish buyers used to select fishers and trust them with fishing equipment, loans, etc. We designed a simple in-depth interview asking fish buyers how they choose fishers with whom to work with, and pre-tested it with a key informant fish buyer and a key informant fisher. We then used a snowball sample approach to contact all fish buyers in the fishing community of Bahía de Kino, an important small-scale fishing community in the Gulf of California, Mexico (see study site section), and where the first author has been studying since 1999, and thus knows some of these fish buyers. Most of the interviews were conducted at the interviewees’ homes. The interviewer generally eased fish buyers into the conversation by first asking them about their personal history, particularly how they got into the business in the first place, before directly asking them how they chose fishers to work with and not to work with. Following this procedure we interviewed all six major fish buyers for one of the key species (pen shells) harvested in the village between May 23 and June 17, 2011 and followed up with interviews with key informants on February 2nd of 2014 after initial interview data had been processed. The main emphasis of the follow-up interviews was on clarifying if the different wording used by each fish buyer referred to different expressions of the same concept of interest to us (e.g., reliability) or not.

### Assembling longitudinal quantitative databases

To understand how constitutional choice plays out under patron-client relationships and cooperatives, we built a longitudinal database of transactions between fishers and fish buyers. With these data, we build a database on the number of repeated interactions with each fisher. An interaction was defined as an exchange between a fish buyer and a fisher and whether a loan was provided and/or a catch was brought. Our goal was to assess the extent to which the interactions in patron-client relationships were repeated and trustworthy in order to gain insight into the process through which stable groups of resource harvesters are constituted. In other words, the pattern of interest for both databases was to empirically identify the different types of transactions emerging from the data, and use these types of transactions to characterize fishers’ behaviors.

As discussed in greater depth in the results section, we identified four patterns of fisher behavior in the data, which we then utilized to categorized the set of interactions between each fish and the patron or cooperative. These characteristic patterns of behavior were labeled as ‘reliable’, ‘less-reliable’, ‘unreliable’, and ‘price-seekers.’ ‘Reliable’ fishers were defined by transactions characterized by loan received and payment made either directly (cash) or indirectly (landed catch). ‘Less-reliable’ only differed from ‘reliable’ in that their track record of transactions with the fish buyer or cooperative was lower as measured by their number of transactions. ‘Unreliable’ fishers constituted instances were fishers had been granted a loan but did not make any repayment to it, and ‘price seekers’ as those that did not ask for a loan but landed a catch.

### Study sites

**Fig 3:**
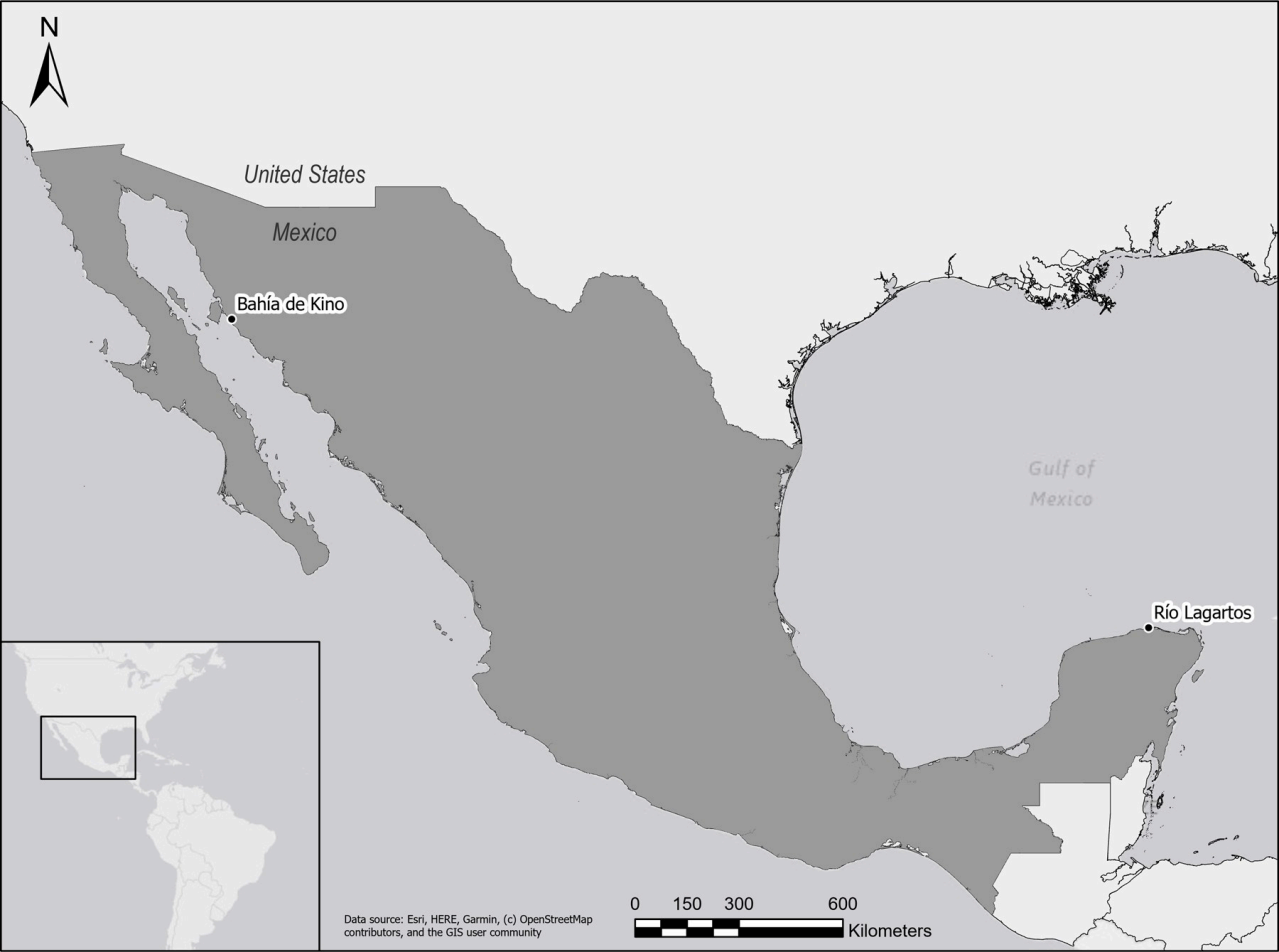
Study sites. The study of patron-client relations was conducted in Bahia de Kino, Sonora and the one about cooperatives in the community of Rio Lagartos, Yucatan, Mexico. Data source: Esri.

Detailed and accurate data on transactions within cooperatives and/or patron-client relationships is rare in small-scale fisheries, so the selection of study sites was guided by data availability. However, the study sites, while geographically distant (Fig 3), share similar characteristics. Bahia de Kino has a population of around 6,000 people, most of whom are dependent directly or indirectly on fishing, and it is estimated there are around 200 boats harvesting 66 species of fish and shellfish throughout the year [38]. Rio Lagartos’s population of ~5,000 people is also highly dependent upon small-scale fishing, with about half of the ~600 fishers working for patron-client relationships and the other half for fishing cooperatives [28]. Fishers in Rio Lagartos also harvest a wide variety of species including snapper/grouper and other finfish, spiny lobster, octopus, and sea cucumber.

#### The patron-client database

To assemble the patron-client database we gained access to the personal lending/payment logbooks belonging to one of the most important fish buyers operating in Bahia de Kino in terms of the value and volume of landings (estimated to be of 25–50% of the total landings in the community). Data encompassed a non-continuous period of roughly five years (April 24, 2003 to February 25, 2009). These log books generally documented transactions on: 1) what species was bought, 2) on what day, 3) from whom, 4) and how much money the fish buyer lent for that particular fishing trip. Logbooks were hand-written and data recorded in a non-systematic fashion. It took more than six months to stitch together six logbooks and digitalize all records. The fish buyer informant clarified any remaining questions related to his note taking quirks. Monetary data amounts about loans and payments for catch was not included due to reliability issues. We could reliably and systematically identify the date, name of each fisher working for the fish buyer, whether a loan was made to him on a particular day, and whether a catch was brought back.

We anonymized the logbooks at the time they were digitalized. Names were replaced with a code that cannot be traced to the original name. Original data is securely stored and personnel conducting analysis have no access to the original data. Once data was plotted (as in Fig 3), we met with the fish buyer informant to discuss data interpretation. This process reassured us the data provided an accurate representation of the types of arrangements developed with fishers during that time period and the interpretation presented in the discussion section.

#### The fishing cooperative database

We gained access to financial records for a fishing cooperative known for keeping adequate accounting and we followed the same anonymization protocol as for the previously described database. This data accounts for a continuous five-year time period from 2009 to 2013.

One notable difference between the two data sets is that in the cooperative dataset, not all loans are repaid in one day’s fish landings. Sometimes, more substantial loans are repaid in installments. As a result, while all fishers categorized as ‘reliable’ in the patron-client data are guaranteed to have fully repaid loans, the same is not true for the cooperative fishers who may show multiple loan repayments without necessarily paying the full balance of their loans. However, given that the purpose of our analysis is to evaluate repeated and trustworthy interactions (constituting relatively stable groups), this difference does not undermine the validity of our empirical analysis. A fisher who is repeatedly making installments is not inconsistent with a reliable fisher. Furthermore, the distinction between ‘reliable’ and ‘less reliable’ fishers (differentiated according to number of loan repayments) allows us to mitigate the risk inherent in our analytical approach of mislabeling a reliable fisher.

## Results

First we report on the interview data that allowed us to identify the fishers’ characteristics sought after by fish buyers when deciding with whom to engage in constitutional choice arrangements. Then we present the longitudinal dataset representing transactions (e.g., the specific expression of their constitutional choice arrangements) between fishers and fish buyers over time.

### Fish buyers seek highly reliable fishers

In interviews fish buyers reported seeking to work with reliable fishers, defined by interviewees as those with one, some, or all of the following characteristics: 1) Fishers who go fishing on a predictable basis when the weather is good, as opposed to not going out (e.g. because of substance abuse or ill-maintained equipment). 2) Fishers who are capable of landing adequate catch because they have the knowledge and skills to do so. 3) Fishers who uphold agreements to land their catch with the fish buyer who provided loans and inputs instead of with another fish buyer offering a better price.

Fish buyers linked their success partly to their ability to identify highly reliable fishers and develop trustworthy relationships with them and to minimize interactions with fishers behaving opportunistically. One fish buyer stated that “most fishers engage in non-trustworthy behavior 20 percent of the time and even more frequently in times of resource abundance!”

Lending money to fishers who fail to bring back catch at all, at the right time, or in sufficient volume such that the buyers cannot fulfill commitments with other clients further up the supply chain can lead to the failure of their business. Therefore, fish buyers seek to form and support a core group of reliable fishers that can provide a constant supply of fish.

Fish buyer interviewees stated that once they have identified a reliable fisher who does what is said to do and brings fish regularly, they will continue to lend money and work with this person as long as they can. A fish buyer informant characterized a reliable fisher as “someone that does not ask you for [a lot of] money, goes to work, and brings you a lot of fish.” Another fish buyer stated that reliable fishers are “responsible, hardworking, come through with their commitments, and learn my working system.” Yet another highlighted that “there are fishers that ask you for work and if one sees they are not responsible you do not lend them for gas, they better go ask someone else, it doesn’t matter that they go with another buyer […] because they are not responsible, they are too much trouble, they are more of a problem than the benefits you get from their catch.” Cooperatives also prefer reliable members, those with a reputation of trustworthiness and positive behavior and often put prospective new members on probation before formally accepting them into the organization.

### Patron-client and cooperatives distribute benefits differently

The goals of building two longitudinal databases, one for a patron-client relationship and another for a cooperative, was threefold: 1) to operationalize the notion of reliability as expressed by transaction instances of landing catch and paying back loans, 2) to observe differences between these two opposing ways of organizing fishing, and 3) to more generally illustrate the importance of taking into account issues of constitutional choice into the analysis of harvesting governance in common-pool resources. Each database operationalized their constitutional choice arrangements, i.e., ‘a transaction’ slightly differently based on available data (see methods section).

**Fig 4:**
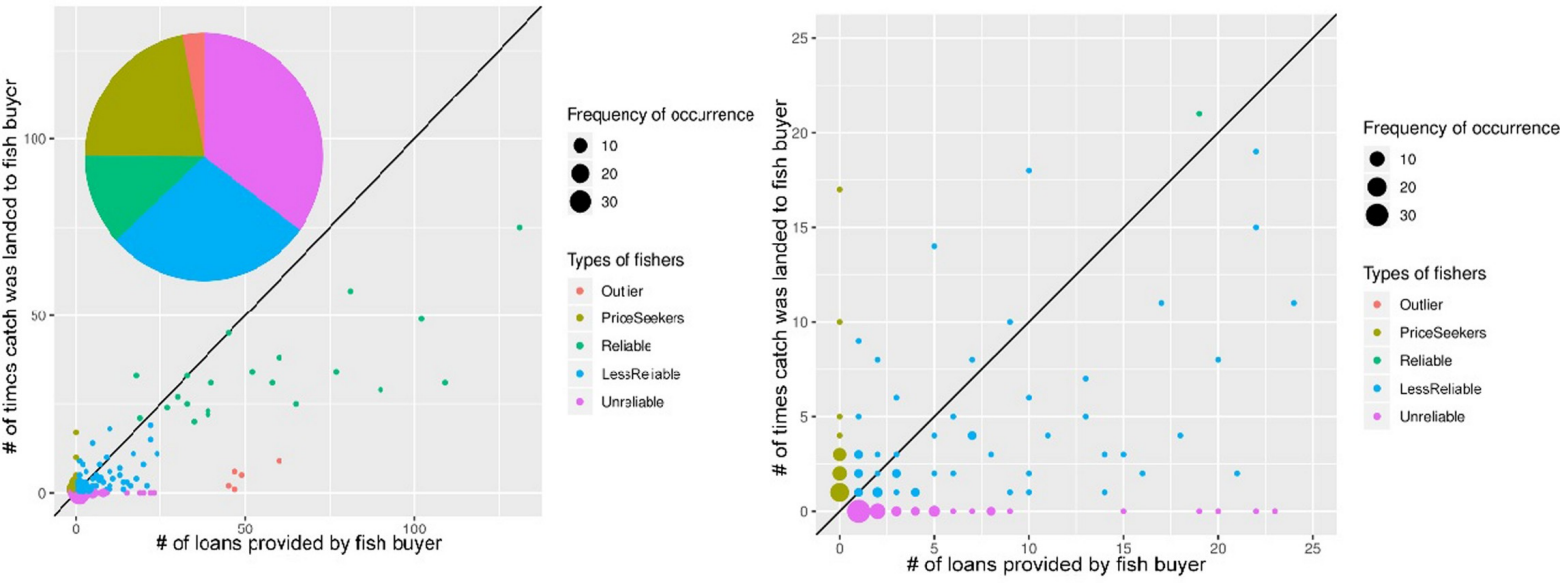
**A** Loan-catch return transactions in a patron-client relationship. Distribution of the four types of fishers interacting with fish buyer: 1) ‘Reliable’ fishers are characterized by a higher number of loan-payment transactions compared to 2) ‘less-reliable’ fishers. 3) ‘Unreliable’ fishers are characterized by no loan repayment, and 4) ‘price seekers’ as fishers who did not received a loan yet landed catch. The 45-degree line indicates the equilibrium between number of loans made and payments received. **B** First 25 loan-catch transactions. The patron-client relationship was characterized by a high number of interactions with fishers who received a loan yet never landed a catch (those on the x axis), illustrating the importance it has for fish buyers to identify reliable fishers to conduct business with.

Fig 4A and 4B show four types of behaviors we identified in the data in terms of fishers’ reliability, measured as a function of the relationship between landed catch and loans granted by the fish buyer. Most fishers (about 80 in the five-year period) working for the patron were given a loan more times than times they landed a catch. A sizable amount never brought a catch and some received multiple loans (labeled as ‘unreliable fishers’). ‘Reliable’ and ‘less-reliable’ fishers brought catch in repeated occasions. ‘Reliable fishers’ are those with the highest number of loans and times they brought catch, and ‘less-reliable’ are those with the lowest levels of number of loans and times brought catch, likely because they have not been working with the fish-buyer for as long as ‘reliable fishers.’ A few fishers also sold catch to the buyer without taking a loan.

**Fig 5:**
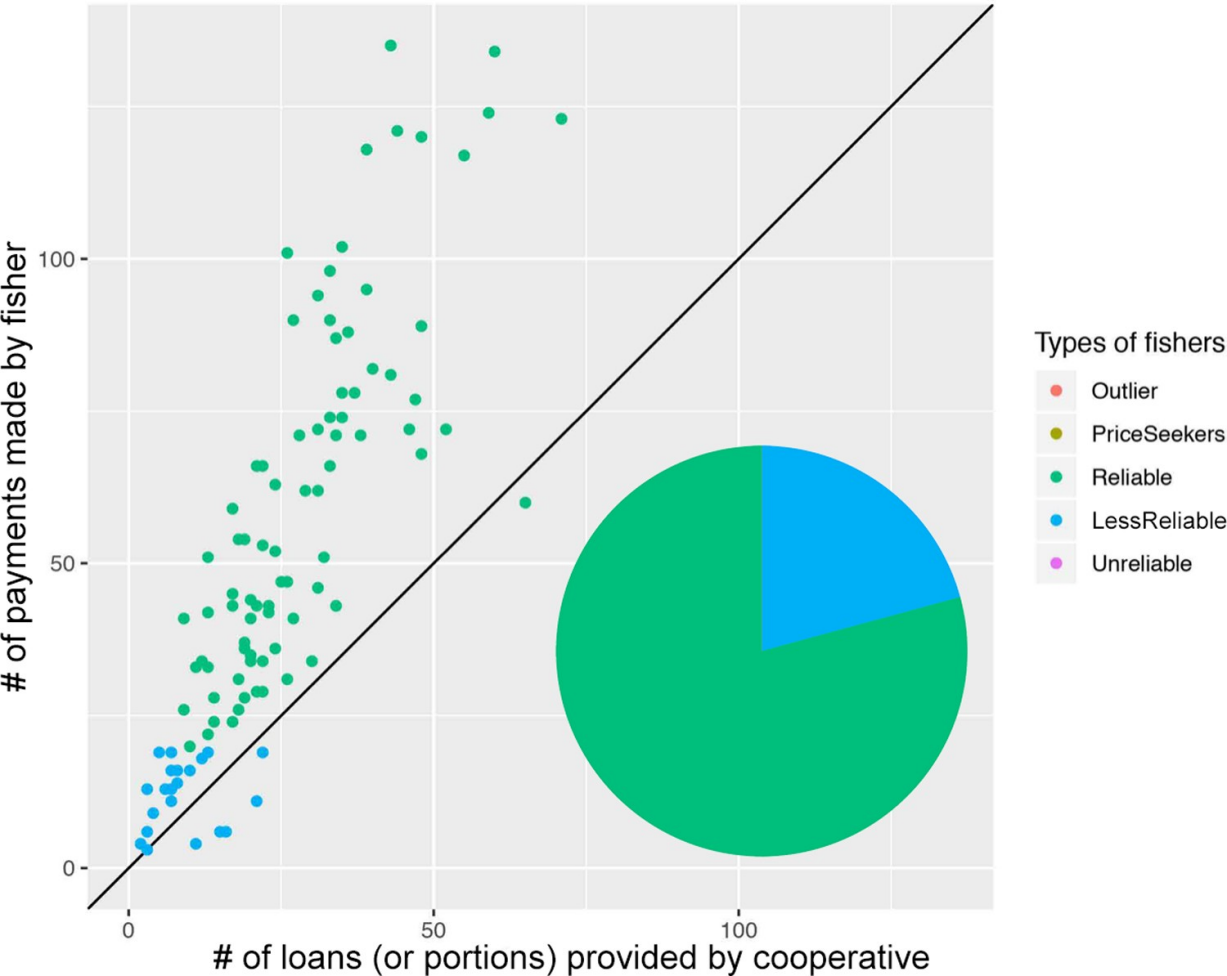
Loan-payment transactions in a cooperative. This dataset shows ‘reliable’ and ‘less-reliable fishers’ but no ‘unreliable’ or ‘price-seeking’ fishers (see Fig 4A for definitions). Because it is common that fishers make partial payments on the same loan (i.e., portion of a same loan), the x-axis should not be read as total number of loans given to a fisher.

Fig 5 shows a contrasting pattern. In the cooperative, the number of loan payments is much higher and almost all fishers made at least a few payments on their loans. ‘Reliable fishers’ are much more abundant and no ‘unreliable’ or ‘price seeker’ fishers were found.

## Discussion

The motivation for this paper emerged from the observation of the overwhelming attention that harvesting has received in the study of common-pool resource governance. While harvesting remains a central action situation to understand collective action dilemmas, our study illustrates one way to incorporate linked action situations influencing harvesting of the commons. Other commons scholars such as Cox [39] and Schlüter et al. [40] have also highlighted the analytical usefulness of linking action situations in other common-pool resource contexts. Here, we endeavored with difficult-to-obtain loan data to illustrate how the process of accessing fishing means of production (through loans) that take place before harvesting in small-scale fisheries is mediated by the organizational choices fishers make (patron-client or fishing associations such as cooperatives).

### Distribution of economic benefits

Overall our data in Figs 4A and 4B and 5 shows that fishers working under a patron-client structure receive less benefits from fishing e.g., are more indebted (measured by the ratio of loan and catch landed) than under the cooperative we examined. The group of “reliable fishers” in Fig 4A are those with whom the patron interacted most frequently (i.e., high number of loans and catch landed). The patron described them as his most trustworthy labor force and saw little risk in giving frequent loans. Overall, under this form of self-governance fishers may be particularly vulnerable to exploitation because they are often in debt to the fish buyer and have no access to collective-choice arenas shaping their working conditions. These findings are consistent with the literature highlighting power differentials and incentives for exploitative relations in patron-client structures [41–42], yet, non-exploitative relationships are possible too [22, 43].

In contrast to the patron-client structure, the cooperative we examined suggests higher levels of loan repayment. Fig 5 shows that most fishers transacting with the cooperative made payments on loans more frequently than they received loans, although because we did not analyze the amount paid in each interaction, it is possible these could be small payments on large loans as it cannot be assumed that one payment equals to one loan. Overall however, fishers working for the cooperative engaged in much more frequent trustworthy behavior toward fulfilling their loans when compared with the fishers who transacted with the patron. In fact, a willingness to provide larger loans would suggest high levels of trust to cooperative fishers who demonstrate high levels of reliability.

The number of unreliable fishers was also much higher in a patron-client structure than in the cooperative. What might account for this? We know this was a well-functioning cooperative, and it is possible that it had the ability to pre-select reliable fishers as subjects of membership. Previous modeling and empirical work have shown cooperatives can build more trust because they cannot exchange their members as frequently as patron-client relationships [44]. Patron-client relationships’ lower transaction costs of entering and exiting the arrangement allows them to accumulate more reliable fishers faster than cooperatives, yet have a lower success rate in keeping reliable fishers within the arrangement than cooperatives [44, 28].

Field experiments have also demonstrated that members of agricultural cooperatives in the Philippines exhibited higher levels of trust and trustworthiness than non-member farmers [45]. Unlike in patron-client relationships where property rights, capital, and financial resources are private goods of the patron, in cooperatives these goods are collectively provided and owned by all the members. As a result, fishers might find more incentives for opportunistic behavior in patron-client relationships because it only violates the trust of a single individual while in a cooperative it can violate the trust of all the members. Furthermore, informal sanctioning of opportunistic behavior is more likely to occur in a cooperative, where all members have an interest in monitoring each other’s behavior than in a patron-client relationship where opportunistic behavior is a direct concern only of the patron himself. Finally, we did not measure a number of demographic factors including familial ties that likely help to account for the large variation we see among subgroups of fishers, whether they are characterized as reliable, less-reliable or unreliable.

### Influence of ‘reliable fishers’

We have argued trust-based interactions generated at the constitutional-choice level between the patron and fishers are essential for this form of organization to persist over time [44]. Yet in patron-client relationships trust-based interactions only can affect operational-level action situations (i.e., harvesting) because fishers usually do not own the fishing means of production, property-rights, or capital. Thus, they do not have access to collective-action arenas in which decisions about harvesting and labor conditions are shaped, which can impact the sustainability of the resource and fishers’ long-term well-being. As a result, they have no recourse to represent and defend themselves when needed. Cinti et al. [38] documented how fishers in Mexico were excluded from participating in local fisheries management because legally, their patrons owned the fishing permits under which they fish, and thus only they could participate in local collective-choice arenas about fishing.

In contrast, in fishing associations like cooperatives, fishers are more likely to be able to participate in shaping operational and collective-choice-level action situations, and so trust and trustworthiness generated at the constitutional-choice level can have broader effects over harvesting and financial distribution of benefits. For the cooperative in our data set, which is constituted by a high number of reliable fishers, we have documented a number of instances in which the cooperative engaged in successful collective-action to monitor access to outsiders to their fishing grounds, negotiate, and lobby the government to defend their fishing rights [46]. Overall, fishers also benefit from developing a trustworthy relationship with their fishing patrons because it increases the likelihood they will also gain access to credit for purposes beyond the fishing arena. Fishers reported having access to loans to fix a boat or motor, buy vehicle parts, medical expenses or payment of credit tabs for groceries and household items at the local store, among others.

The need to find reliable fishers to work with also affects harvesting patterns and fisheries sustainability. When fish buyers or cooperatives do not find reliable fishers in the locality to work with, they face incentives to bring fishers from elsewhere, effectively increasing fishing pressure and the risk of overfishing. The movement of fishers is a persistent source of conflict in fishing and has been extensively documented [47, 48]. In the Yucatán Peninsula in Mexico, migratory labor can threaten the resilience of well-organized fishing associations, by stressing local fishers’ ability to control access of migrant fishers to their common-pool resources [28, 46], as well as the provision of other public goods like health and schooling available in rural coastal communities.

We should also point to the unrealistic expectation that anyone can be reliable 100% of the time.

In competitive labor markets, fishers might find strong incentives to change their loyalty depending on the working conditions being offered. Even in settings like small-communities where oligopolies can easily be formed by fish patrons, changes in abundance and constant demand, compel buyers to compete some of the time. A fish buyer who is too strict about loan repayment may lose reliable fishers, ultimately reducing fish supply. Furthermore, by allowing fishers who sometimes fail to repay loans to continue working with the fish buyer, the fish buyer may be able to extend fishers’ obligations and foster more stable buyer-seller relationships. In interviews fish buyers acknowledged that periodic unreliability is part of the costs of doing business, especially in situations where fishers face strong incentives to behave opportunistically, for example, in the case of income shortfalls due to a family illness.

## Conclusion

Our study makes the case to broaden the analytical scope beyond-harvesting to achieve a fuller understanding of the governance of the commons. We argue that institutional analysis concepts such as *linked action situations* and *constitutional-choice level*, can be readily deployed.

Our empirical study in Mexico illustrated how linking *constitutional* to operational level and collective-choice activities allows incorporating pre-harvesting choices shaping how harvesting activities take place such as access to credit, fishing means of production, or choice of organizational structure. These collective action processes have not received enough attention in the literature of fishing common-pool resources, yet they shape overall outcomes, particularly in terms of the distribution of benefits generated by harvesting and fishers’ well-being. Our call to extend the analysis of common-pool resources as networks of action situations makes explicit the need to consider action situations where negotiations of access to inputs of production are being conducted, so that their interaction with operational level harvesting activities are better accounted for. Our work resonates with Elinor Ostrom’s call to increase attention to the interactions resulting from different action situations [49, 50], which has not been sufficiently fulfilled to date. We expand the application of the concept of the constitutional arena to the study of informal fishing associations, which has also received less attention in the literature and make more visible the importance of accounting for factors such as reliability, trust, and trustworthiness that drive group formation [43] and shape actors’ incentives and capabilities with regard to their engagement in operational and collective choice arenas.

In sum, we offered here two examples of conceptual strategies to more directly incorporate decisions around harvesting practices are influenced by how fishers organize to gain access to capital, labor, and commercialization, which have always been key aspects of concern for common-pool resources governance and scholarship, but are not effectively incorporated when only focused on harvesting as the main analytical concern.

## Supporting information

S1 Data (XLSX)
